# Construction and evaluation of DNA vaccine encoding Crimean Congo hemorrhagic fever virus nucleocapsid protein, glycoprotein N-terminal and C-terminal fused with LAMP1

**DOI:** 10.3389/fcimb.2023.1121163

**Published:** 2023-03-21

**Authors:** Yong-Liang Hu, Lian-Qing Zhang, Xiao-Qian Liu, Wei Ye, Yue-Xi Zhao, Liang Zhang, Zun-Xian Qiang, Lin-Xuan Zhang, Ying-Feng Lei, Dong-Bo Jiang, Lin-Feng Cheng, Fang-Lin Zhang

**Affiliations:** ^1^ Department of Microbiology, Air Force Medical University (The Fourth Military Medical University), Xi’an, China; ^2^ Department of Dermatology, The Eighth Medical Center of PLA General Hospital, Beijing, China; ^3^ College of Life Sciences, Northwest University, Xi’an, China; ^4^ School of Medical Technology, Shaanxi University of Chinese Medicine, Xianyang, China; ^5^ Department of Immunology, Air Force Medical University (The Fourth Military Medical University), Xi’an, China

**Keywords:** Crimean-Congo hemorrhagic fever virus, DNA vaccine, lysosome associated membrane protein 1, transcription and entry-competent virus-like particles, humanized transgenic mice

## Abstract

Crimean-Congo hemorrhagic fever virus (CCHFV) can cause severe hemorrhagic fever in humans and is mainly transmitted by ticks. There is no effective vaccine for Crimean-Congo hemorrhagic fever (CCHF) at present. We developed three DNA vaccines encoding CCHFV nucleocapsid protein (NP), glycoprotein N-terminal (Gn) and C-terminal (Gc) fused with lysosome-associated membrane protein 1 (LAMP1) and assessed their immunogenicity and protective efficacy in a human MHC (HLA-A11/DR1) transgenic mouse model. The mice that were vaccinated three times with pVAX-LAMP1-CCHFV-NP induced balanced Th1 and Th2 responses and could most effectively protect mice from CCHFV transcription and entry-competent virus-like particles (tecVLPs) infection. The mice vaccinated with pVAX-LAMP1-CCHFV-Gc mainly elicited specific anti-Gc and neutralizing antibodies and provided a certain protection from CCHFV tecVLPs infection, but the protective efficacy was less than that of pVAX-LAMP1-CCHFV-NP. The mice vaccinated with pVAX-LAMP1-CCHFV-Gn only elicited specific anti-Gn antibodies and could not provide sufficient protection from CCHFV tecVLPs infection. These results suggest that pVAX-LAMP1-CCHFV-NP would be a potential and powerful candidate vaccine for CCHFV.

## 1 Introduction

Crimean-Congo hemorrhagic fever (CCHF) is a severe febrile disease in humans, the mortality rate of which can reach 30% (Tipih and Burt, 2020). It is caused by Crimean-Congo hemorrhagic fever virus (CCHFV), a negative-sense RNA virus in the Bunyavirales order, Nairoviridae family, Orthonairovirus genus. CCHF is transmitted to humans through tick infection or close contact with the body fluids of an infected person or animal. The disease is widely distributed in Asia, Africa, the Middle East, and Eastern Europe, which is consistent with the wide geographic distribution of tick vectors of the Hyalomma species (Aligholipour Farzani et al., 2019a). In addition, global climate change may lead to the expansion of the Hyalomma tick, thus introducing CCHFV into new areas (Hawman et al., 2021). At present, treatment options for CCHFV are limited, including supportive treatment options, such as serum and platelet transfusion. Ribavirin has also achieved success in hospitalized patients during the early stage of clinical illness, but human clinical and animal experimental data provide contradictory evidence for the benefits of ribavirin in treating CCHFV and indicate that better treatment is needed (Berber et al., 2021). Therefore, a safe and protective vaccine is needed to prevent disease and control the spread of CCHFV among the public.

Despite having no globally licensed CCHFV vaccine, a single vaccine has been used in Bulgaria since 1974. This vaccine is produced from mouse brain that is inactivated with chloroform. This vaccine is unlikely to obtain international approval because neural tissue content may cause autoimmune and allergic reactions (Mousavi-Jazi et al., 2012). Researchers have evaluated several vaccine platforms in mouse models, including subunit-based (Kortekaas et al., 2015), plant-based (Skarjinskaia et al., 2013), virus-like replicon particle-based (Devignot et al., 2015), DNA-based (Hinkula et al., 2017), mRNA-based (Aligholipour Farzani et al., 2019a) and viral vector-based vaccines (Dowall et al., 2016; Rodriguez et al., 2019; Aligholipour Farzani et al., 2019b), with varying efficacies from complete to no protection. However, the factors associated with CCHFV vaccine protection remain unclear. It is worth noting that the contribution of neutralizing antibodies to vaccine-mediated protection appears to be dispensable (Aligholipour Farzani et al., 2019c). This suggests that the ability to stimulate specific cellular immune responses, such as cytotoxic T-cell (CTL) activation, may be crucial for the CCHFV vaccine.

Many previous studies have shown that DNA vaccines mainly encode endogenous antigens that are processed to form class I peptides/major histocompatibility complexes (MHC I), which primarily initiate CTL activation (Jiang et al., 2015). Previously, we constructed a chimeric DNA vaccine in the pVAX1 plasmid that encodes the fusion protein of Hantaan virus (HTNV) Gn and lysosome-associated membrane protein 1 (LAMP1), and the results showed that pVAX-LAMP1/HTNV-Gn significantly enhanced HTNV-specific immune responses and protection after consecutive immunizations in a short period (Jiang et al., 2015; Jiang et al., 2017; Jiang et al., 2018).

Lysosome-associated membrane protein 1 (LAMP1) is a highly glycosylated glycoprotein belonging to the LAMP family. LAMP1 is mainly located in endosome-lysosome membranes. LAMP1 can assist protein degradation *via* the lysosome pathway and antigen presentation *via* MHC II molecules. We hope that by fusion expression of LAMP1 and CCHFV-related antigen proteins, the degradation of the antigen proteins will be promoted through the lysosome pathway, and the antigen presentation efficiency of CCHFV-related proteins will be improved, thus improving its immunogenicity. Similar to HTNV, CCHFV and other viruses in the Bunyavirales order have three negative sense, single-stranded RNA segments, large (L), medium (M) and small (S), which encode the RNA-dependent RNA polymerase, envelope glycoprotein N-terminal (Gn) and C-terminal (Gc), and nucleocapsid protein (NP), respectively. At present, many CCHFV vaccine studies have largely focused on Gn, Gc and NP (Tipih and Burt, 2020). Therefore, our research plans to construct a variety of novel CCHFV DNA vaccines (pVAX-LAMP1-CCHFV-Gn, pVAX-LAMP1-CCHFV-Gc, pVAX-LAMP1-CCHFV-NP) and evaluate their immunogenicity and protective efficacy.

Animal models have always been an important topic in the field of CCHFV vaccine research. One kind of more economical laboratory animals that better mimic the human immune response is a common goal. The human HLA-A11/DR1 transgenic mice used in this research were constructed by inserting human HLA-A11 (MHC-I) and HLA-DR1 (MHC-II) genes into the wild-type C57BL/6 mouse genome. The HLA-A11 and HLA-DR1 genes are the most common human genotypes. Approximately 20%-30% of the Chinese population are of this genotype. In addition, 10%-15% of Europeans also have this genotype. Therefore, the transgenic mice could theoretically partially simulate the antigen-presentation process of vaccines in humans.

## 2 Materials and methods

### 2.1 Plasmids and cells

The plasmid pVAX-LAMP1 was constructed as previously described (Jiang et al., 2015). The related plasmids (including pSMART-LCK-vL-NanoLuc, pCAGGS-LCK-L, pCAGGS-S, pCAGGS-M and pCAGGS-T7) used in this study to construct CCHFV tec-VLP were provided by Professor Eric Bergeron (the Centers for Disease Control and Prevention of the United States of America, Atlanta, Georgia, USA.) (Zivcec et al., 2015). HeLa, 293T, and Huh7 cells were purchased from ATCC (Rockville, MD, USA) and maintained in DMEM (Corning, Nanjing, Jiangsu, China) supplemented with 10% (v/v) FBS (Sigma-Aldrich, St. Louis, MO, USA) at 37°C with 5% CO_2_. Mouse splenocytes and peritoneal macrophages were collected from human MHC (HLA-A11/DR1) transgenic mice (Zeng et al., 2016) and maintained in RPMI-1640 medium (Corning, Nanjing, Jiangsu, China) supplemented with 10% (v/v) FBS at 37°C with 5% CO_2_.

### 2.2 Antibodies and proteins

Anti-CCHFV NP and Gc rabbit polyclonal antibodies (pAb) were purchased from Integrated BioTherapeutics, Inc. (Gaithersburg, MD, USA). Anti-CCHFV Gn rabbit pAb was purchased from GenScript company (Nanjing, Jiangsu, China). The Sp2/0 mAb was supplied by our laboratory. Flow cytometry (FCM) antibodies, including APC/Cyanine7 anti-mouse CD3, FITC anti-mouse CD4, PE/Cyanine7 anti-mouse CD8, PE anti-mouse IL-4, and Brilliant Violet 421™ anti-mouse IFN-γ, were purchased from Biolegend, Inc. (San Diego, CA, USA). Other secondary antibodies, including FITC-conjugated and HRP-conjugated antibodies, were purchased from Proteintech Group, Inc. (Wuhan, Hubei, China). Purified CCHFV Gn and CCHFV NP were purchased from Abcam (Boston, MA, USA), and purified CCHFV Gc was purchased from The Native Antigen Company (Kidlington, Oxford, UK).

### 2.3 CCHFV transcription and entry-competent virus-like particles

Preparation of CCHFV tecVLPs: Huh7 cells were inoculated on 6-well plates and cultured in CO_2_ incubator at 37°C until the cell confluence was approximately 70%-80%. The following plasmids were cotransfected: pSMART-LCK-vL-NanoLuc 100 ng, pCAGGS-LCK-L 800 ng, pCAGGS-S 400 ng, pCAGGS-M 400 ng, pCAGGS-T7 200 ng. 16-18 h after transfection, the liquid in the bottle was removed, the cells in the bottle were gently washed with DPBS, and fresh medium was added. After the cells were cultured at 37°C for 3 days, the cell supernatant was collected and centrifuged at 4°C at 12000 r/min for 5 min. After centrifugation, the cell debris was discarded. The supernatants were filtered through a 0.45 µm filter. After sorting, the fragments were stored at -80°C.

In this study, the Karber method was used to detect the TCID_50_ of CCHFV tecVLPs. The methods are as follows: The CCHFV tecVLPs stock solution was successively diluted in a 10-fold gradient, and then Huh7 cells were inoculated in 48-well cell culture plates. When the cell confluence was approximately 70%-80%, the helper plasmids PCAGGS-LCK-L 200 ng and PCAGGS-S 100 ng were pre-transfected. After transfection for 24 h, 100 µL/well of CCHFV tecVLPs diluted at each gradient were added to cells with 8 wells for each dilution. After 2 h of infection, fresh medium was replaced for further culture. After 48 h of infection, the cell lysate was collected, and NanoLuc expression was detected. The results were recorded, and the TCID_50_ of CCHFV tecVLP was calculated according to the formula below.

lgTCID_50_ =L+D×(S-0.5)

L: logarithm of the highest dilution; D: difference between logarithms of dilution; S: summation of positive well ratios.

### 2.4 Animals

Adult female human MHC (HLA-A11/DR1) transgenic mice (8 weeks old) were provided by the State Key Laboratory of Pathogen and Biosecurity (Beijing, China) (Zeng et al., 2016). All animal experiments were performed according to the protocols approved by the Air Force Medical University Animal Experimental Welfare & Ethical Committee (Xi’an, Shaanxi, China; approval no. 20210396).

### 2.5 Construction and identification of recombinant plasmids

CCHFV IbAr10200 GPC (GenBank ID AF467768.2) and NP (GenBank ID U88410.1) gene fragments were synthesized by Sangon Biotech (Shanghai, China). cDNAs encoding CCHFV Gc, Gn and NP were obtained by PCR and then cloned into the plasmids pVAX1 and pVAX-LAMP1. LAMP1 (GenBank ID BC021288.2) gene fragments had already been inserted into the plasmid pVAX-LAMP1 (plasmid map shown in **Supplementary Figure 1**). All recombinant plasmids were confirmed by PCR amplification and DNA sequencing by Sangon Biotech. The primers for PCR are provided in **Supplementary Table 1**. The recombinant plasmids were transfected into 293T cells. The transfected cells were collected and lysed 48 h after transfection. The protein samples were subjected to sodium dodecyl sulfate-polyacrylamide gel electrophoresis (SDS-PAGE). Then, the proteins in the gel were transferred to a PVDF membrane. The membranes were blocked with blocking buffer and incubated with specific primary antibodies and corresponding secondary antibodies. Finally, ECL substrate solution was added, and the protein signals on the membrane were detected and analyzed. The recombinant plasmid vectors were also transfected into HeLa cells. Cells were cultured in 24-well plates with a sterile glass slide in each well. After 48 h of transfection, cells on the glass slides were fixed with 4% paraformaldehyde for 15 min and washed 3 times with PBS. Then, the cells were permeabilized for 15 min with 0.5% Triton X-100. After 3 washes with PBS, the cell slides were blocked with 3% BSA-PBS solution for 30 min at room temperature. Specific antibodies (anti-CCHFV Gc, Gn or NP) were incubated with the cells on slides at 4°C overnight. FITC-conjugated goat anti-rabbit antibody was used as a secondary antibody. Hoechst 33258 was used for nuclear staining at room temperature for 10 min in the dark. Finally, the cells on the glass slides were observed under a fluorescence microscope.

### 2.6 Immunizations

Female human MHC (HLA-A11/DR1) transgenic mice were divided into 9 groups (10 mice per group) (**Supplementary Table 2**). The mice were injected intramuscularly with 70 µg/200 µL of either plasmid per mouse. All immunizations were performed three times with a 3-week interval. Three weeks after the completion of the third immunization, sera were collected by retroorbital plexus puncture (from 5 mice per group). The sera obtained were used to detect specific antibodies for each antigen and neutralizing antibodies in the sera. Meanwhile, the splenic cells of mice were extracted for subsequent experiments. The extracted splenic cells will be used to detect the secretion of cytokines, the cytotoxic effects of splenic cells, and the proportion of different types of splenic lymphocytes. The above experiments were used to detect the immune effects after immunization with DNA recombinant vectors. In addition, the remaining five mice in each group were used for the CCHFV tecVLPs challenge assay to evaluate the *in vivo* immune protection effect.

### 2.7 Detection of CCHFV NP-, Gc- and Gn-specific antibodies

The titers of CCHFV NP-, Gc- and Gn-specific antibodies in the sera were detected by ELISA. Viral antigen proteins, including CCHFV NP, Gc and Gn, were used as coated antigens. The coated antigen concentration was 5 µg/mL and 100 µL/well. The plates were blocked with blocking solution containing 1% BSA in PBST. Serial dilutions (1:100, 1:200, 1:400, 1:800, 1:1600, 1:3200) of sera from the immunized mice were added to the plates to react with the corresponding antigen. Commercial antibodies were diluted according to the manufacturer’s instructions and used as positive controls. The Sp2/0 mAb was used as the negative control antibody. HRP-conjugated goat anti-mouse antibody and HRP-conjugated goat anti-rabbit antibody were used as secondary antibodies for detection. The primary and secondary antibody diluents were universal antibody diluents (Sangon Biotech, Shanghai, China). A 450 nm wavelength absorbance >0.1 and a positive/negative (P/N) value > 2.1 were considered significant.

### 2.8 Microneutralization test

Here, we used CCHFV tecVLPs system instead of CCHFV to participate in the microneutralization test. The construction of tecVLPs and the establishment of the Huh7 cell infection model are described in the 2.3. CCHFV tecVLP and supplementary data (**Supplementary Figures 2-4**). The microneutralization test was performed on monolayers of Huh7 cells grown in a 96-well cell culture plate. Cells were plated at a density of 2×10^4^ cells per well for 18–24 h before testing. when the degree of cell density was approximately 70%–80%. Four hours after transfection, the medium of the cells was changed to 2% DMEM. The cells were incubated in a CO_2_ incubator at 37 °C for 24 h. The sera from the immunized mice were filtered through 0.22 µm filters, serially diluted twofold from an initial 1:10 dilution to 1:320 in PBS and combined with an equal volume of 100 TCID_50_ CCHFV tecVLPs. After 90 min, the mixture was transferred to monolayers of Huh7 cells and incubated at 37°C for 2 h in a 5% CO_2_ incubator. Then, the medium of the cells was changed to 2% DMEM. After 3 days, the cells were collected and lysed. The Nano-Luc activities were detected using the Nano-Glo™ Luciferase Assay System (Promega, Madison, WI, USA). The neutralizing antibody titers were defined as the maximum dilution of serum that inhibited CCHFV tecVLPs infection in 50% of the cells.

### 2.9 Detection of cytokines secreted from splenocytes

An ELISPOT assay was performed to detect cytokines, including IFN-γ, IL-2, IL-4 and IL-10, secreted by splenic cells with a Mabtech ELISPOT kit (Stockholm, Sweden). Briefly, 96-well ELISPOT plates were precoated with anti-IFN-γ, anti-IL-2, anti-IL-4, and anti-IL-10 mAbs. Splenocytes (1×10^6^ cells in 100 µL) were added to each well and stimulated with a mixture of CCHFV-purified Gn, Gc and NP (10 µg/mL) or with the positive mitogenic stimulator concanavalin A (ConA, 4 µg/mL). The plates were incubated at 37 °C and 5% CO_2_ for 24 h, followed by streptavidin-HRP for 1 h at room temperature. Then, 100 µL of substrate was added, and the plates were washed with deionized water and air-dried at room temperature. The spots were counted using an ELISPOT reader system (AID, Strasberg, Germany). The results were expressed as the mean number of specific spot-forming cells per 1×10^6^ splenocytes.

### 2.10 Cytotoxicity assay

A CytoTox 96™ nonradioactive cytotoxicity assay kit (Promega, Madison, WI, USA) was used according to the manufacturer’s instructions to detect the level of antigen-specific cytotoxic T lymphocytes (effect cells) in response to the target cells (peritoneal macrophages from human MHC (HLA-A11/DR1) transgenic mice cotransfected with pVAX-CCHFV-Gn, pVAX-CCHFV-Gc, pVAX-CCHFV-NP) in different groups. The target cells were plated onto 96-well U-bottomed microtiter plates at 2×10^4^ cells/50 µL per well. The splenocytes from the immunized mice were added to a final volume of 50 µL with effector/target (E/T) ratios of 100:1, 50:1, and 20:1. Normal splenocytes were added as a negative control. The test plate included the following cells as controls: spontaneous release of lactate dehydrogenase (LDH) by effector cells (50µL effector cells and 50µL medium), spontaneous release of LDH by target cells (50µL target cells and 50µL medium), maximum LDH release by target cells (50µL target cells, 50µL medium and 10µL cell lysis buffer), volume correction control (100µL medium and 10µL cell lysis buffer), medium background control (100µL medium). The mortality of target cells was calculated as follows: % cytotoxicity = [(E-St-Se)/(M-St)]×100 (E, LDH release amount of effector target cells; St, the target cells spontaneously release LDH; Se, spontaneous release of LDH by effector cells; M, maximum LDH release from target cells).

### 2.11 Flow cytometry

Approximately 1.0×10^7^ splenic cells were taken from each mouse and stimulated with the corresponding CCHFV antigen protein for 24 h, and protein transport inhibitors were added 8 h before testing. Cells were centrifuged at room temperature at 350×g for 5 min to collect cells. The splenocytes of immunized mice were permeated and fixed. Then, the cells were stained with fluorescent primary antibodies (APC/Cyanine7 anti-mouse CD3, FITC anti-mouse CD4, PE/Cyanine7 anti-mouse CD8, PE anti-mouse IL-4, Brilliant Violet 421™ anti-mouse IFN-γ) and incubated at room temperature for 1 h away from light. Cells were suspended in a 500 µL PBS containing cell membrane permeating solution, centrifuged at room temperature 350×g for 5 min, and washed 3 times. After the last wash, the supernatant was discarded, and the bottom of the tube was flicked until the cells were completely separated. Finally, the cells were harvested and analyzed by fluorescence-activated cell sorting using a Beckman Epics XL instrument (Fullerton, CA, USA).

### 2.12 CCHFV tecVLPs challenge of mice

Here, we used the CCHFV tecVLPs system instead of CCHFV to participate in animal protection experiments. The establishment of the HLA-A11/DR1 mouse infection model is described in the supplementary data (**Supplementary Figure 5**). The remaining 5 mice in each group were infected with 100 TCID_50_ CCHFV tecVLPs by intraperitoneal injection at 3 weeks after the last immunization. Three days after CCHFV tecVLPs infection, the mice were killed by cervical dislocation, and tissues (including liver, kidney, spleen, lung, cerebrum and heart) were collected and weighed from these mice, diluted in PBS, and then freeze−thawed (-80°C/37°C) three times after being ground to prepare 10% (g/mL) tissue suspensions. The samples were centrifuged at 12,000 rpm for 30 min at 4 °C, and the supernatants were collected. NanoLuc activities were measured in a Nano-Glo™ Luciferase Assay System.

### 2.13 Statistical analysis

Statistical analyses were performed using GraphPad Prism v.8.0.2 (GraphPad Software, San Diego, CA). To compare the means of two groups, a two-tailed Student’s t test was performed. One-way ANOVA was used to determine statistically significant differences among three or more groups. All data are presented as the mean ± S.D. A significant difference was defined as p < 0.05.

## 3 Results

### 3.1 Construction and expression of the recombinant plasmids

**Figure 1:**
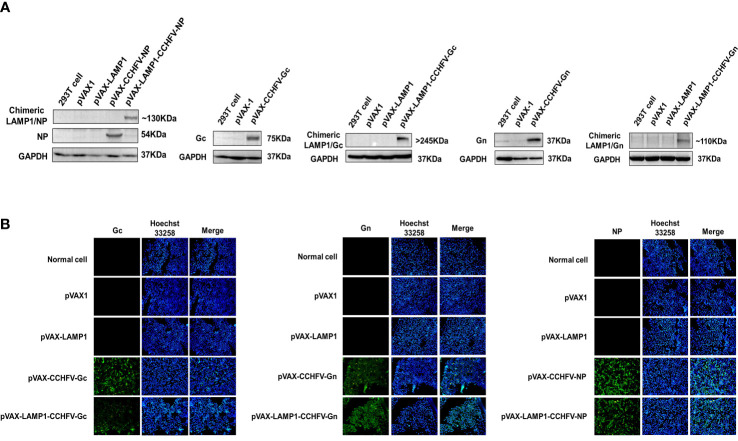
Identification of target protein expression of the recombinant plasmids. **(A)** Western blot analysis of the target protein expression of the recombinant plasmids in 293T cells. **(B)** Immunofluorescence analysis of the target protein expression of the recombinant plasmids in HeLa cells (200×).

To construct several CCHFV-related recombinant expression vectors, the gene fragments of CCHFV NP, Gc and Gn were inserted into pVAX1 and pVAX-LAMP1 to construct recombinant plasmids. Then, we verified these recombinant plasmids by PCR and gene sequencing. To confirm whether the recombinant plasmids could express target proteins successfully, we transfected the recombinant plasmids into 293T cells and HeLa cells and then detected protein expression by western blotting and immunofluorescence, respectively (**Figure 1**). The results showed that the recombinant plasmids successfully expressed the target proteins.

### 3.2 Evaluation of humoral immune responses in the immunized mice

**Figure 2:**
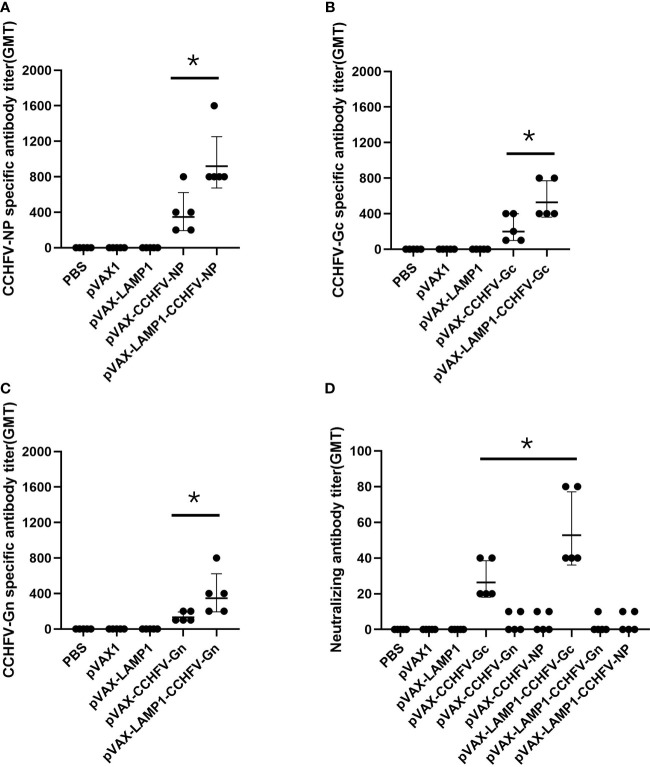
Detection of specific antibodies and neutralizing antibodies in the sera of immunized mice. Mouse sera were collected as described in the Materials and Methods section. The horizontal lines denote the GMT values. **(A)** NP-specific antibody titers were detected using purified NP. **(B)** Gc-specific antibody titers were detected using purified Gc. **(C)** Gn-specific antibody titers were detected using purified Gn. **(D)** Neutralizing antibody titers were detected by determining the ability of the sera to neutralize the CCHFV tecVLPs. (* P < 0.05).

After immunizing mice with recombinant expression vectors, we assessed the serum levels of corresponding specific antibodies. The sera of immunized mice were collected individually and used to detect the titers of CCHFV NP-, Gc- or Gn-specific antibodies by ELISA. The GMTs of the NP-specific antibodies in the sera from the mice immunized with pVAX-LAMP1-CCHFV-NP and pVAX-CCHFV-NP were 916.7 and 343.3, respectively (**Figure 2A**). The GMTs of the Gc-specific antibodies in the sera from the mice immunized with pVAX-LAMP1-CCHFV-Gc and pVAX-CCHFV-Gc were 526.6 and 196.7, respectively (**Figure 2B**). The GMTs of the Gn-specific antibodies in the sera from the mice immunized with pVAX-LAMP1-CCHFV-Gn and pVAX-CCHFV-Gn were 345.1 and 128.3, respectively (**Figure 2C**). The results demonstrated that each recombinant plasmid could enhance the specific antibody response against the CCHFV major antigens NP (p = 0.02), Gc (p = 0.027) or Gn (p = 0.049) after being incorporated with LAMP1, and the GMT of the NP-specific antibodies in the sera from the mice immunized with pVAX-LAMP1-CCHFV-NP was significantly higher than that in any other group.

We then evaluated the neutralizing activity of the induced antibody by detecting the ability of the sera to neutralize CCHFV tecVLPs. The GMTs of the neutralizing antibodies in the sera from the mice immunized with pVAX-LAMP1-CCHFV-Gc and pVAX-CCHFV-Gc were 52.5 and 26.2, respectively. The results also demonstrated that LAMP1 could enhance the neutralizing antibody response (p = 0.033). However, the GMTs of the neutralizing antibodies in the sera from the other groups were extremely low (GMTs < 10; **Figure 2D**). The results indicated that LAMP1 could significantly improve the specific humoral immune response of animals immunized with DNA recombinant vectors. Both the specific antibody titers and the neutralizing antibody titers were significantly improved.

### 3.3 Evaluation of cellular immune responses in the immunized mice

**Figure 3:**
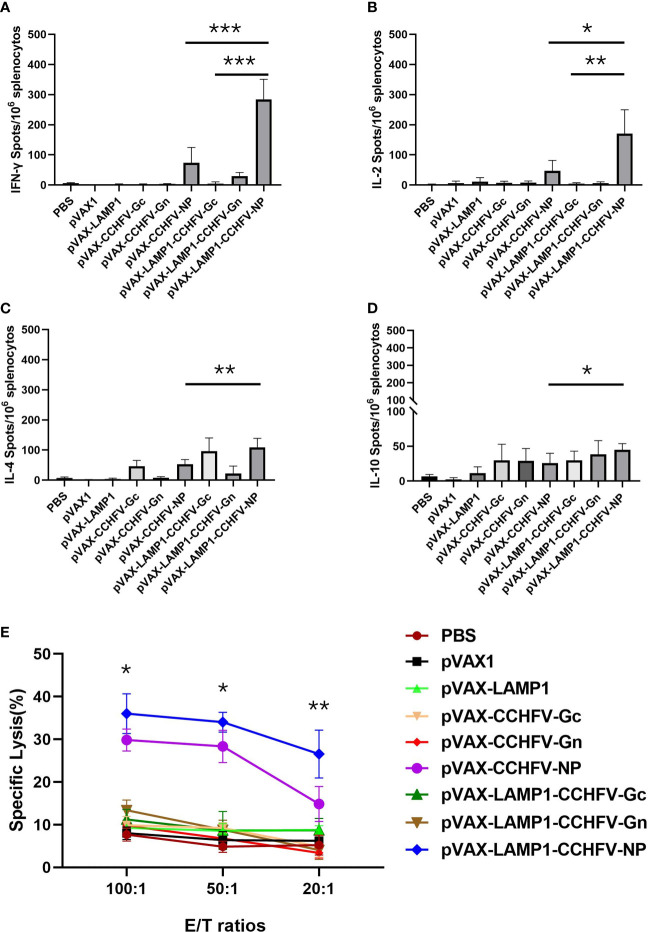
Detection of cytokine secretion and cytotoxicity from the splenocytes of immunized mice. Mouse splenocytes were collected as described in the Materials and Methods section. The results are expressed as the mean value ± SD of five independent experiments. **(A)** ELISPOT analysis of IFN-γ secreted by splenocytes stimulated with a mixture of NP, Gc and Gn. **(B)** ELISPOT analysis of IL-2 secreted by splenocytes stimulated with a mixture of NP, Gc and Gn. **(C)** ELISPOT analysis of IL-4 secreted by splenocytes stimulated with a mixture of NP, Gc and Gn. **(D)** ELISPOT analysis of IL-10 secreted by splenocytes stimulated with a mixture of NP, Gc and Gn. **(E)** Cytotoxicity assay of splenocytes stimulated with a mixture of NP, Gc and Gn. (* P < 0.05, ** P < 0.01, *** P < 0.001).

To evaluate the induction levels of major immune factors in splenic cells in mice immunized with recombinant expression vectors. The secretion of immune factors could reflect the level of cellular immunity to some extent. We examined the immune factors produced by the splenocytes of the immunized mice by the ELISPOT assay. The splenocytes from the mice immunized with pVAX-LAMP1-CCHFV-NP exhibited higher numbers of IFN-γ, IL-2, IL-4 and IL-10 spots compared to the pVAX-CCHFV-NP group (p = 0.0005 for IFN-γ, p = 0.012 for IL-2, p = 0.007 for IL-4 and p = 0.033 for IL-10) and other controls. The splenocytes from the mice immunized with pVAX-LAMP1-CCHFV-NP also exhibited higher numbers of IFN-γ and IL-2 spots than the pVAX-LAMP1-CCHFV-Gc group (p = 0.0007 for IFN-γ and p = 0.009 for IL-2), but there was no significant difference in the numbers of IL-4 and IL-10 (p = 0.625 for IL-4 and p = 0.066 for IL-10). The splenocytes from the mice immunized with pVAX-LAMP1-CCHFV-Gn only exhibited a certain amount of IL-10 (**Figures 3A-D**). LDH release from target cells killed by vaccination-activated splenocytes was measured using a Cytotox 96™ nonradioactive cytotoxicity assay kit. The cytotoxicity of splenocytes from mice immunized with pVAX-LAMP1-CCHFV-NP was enhanced in accordance with the E/T ratio, which was the most significant at the 100:1 ratio. The cytotoxicity of splenocytes from mice immunized with pVAX-LAMP1-CCHFV-NP also showed higher specific cytotoxic activity compared to the pVAX-CCHFV-NP group with E/T ratios of 100:1 (p = 0.031), 50:1 (p = 0.021), and 20:1 (p = 0.0053). Other groups did not show obvious specific cytotoxic activity at any E/T ratio (**Figure 3E**). The comparison of the results of pVAX-LAMP1-CCHFV-NP and pVAX-CCHFV-NP showed that the introduction of LAMP1 significantly improved the ability of NP to induce a specific cellular immune response in mice, suggesting the role of LAMP1 in enhancing antigen immunogenicity.

### 3.4 Identification of the types of splenic T lymphocytes

**Figure 4:**
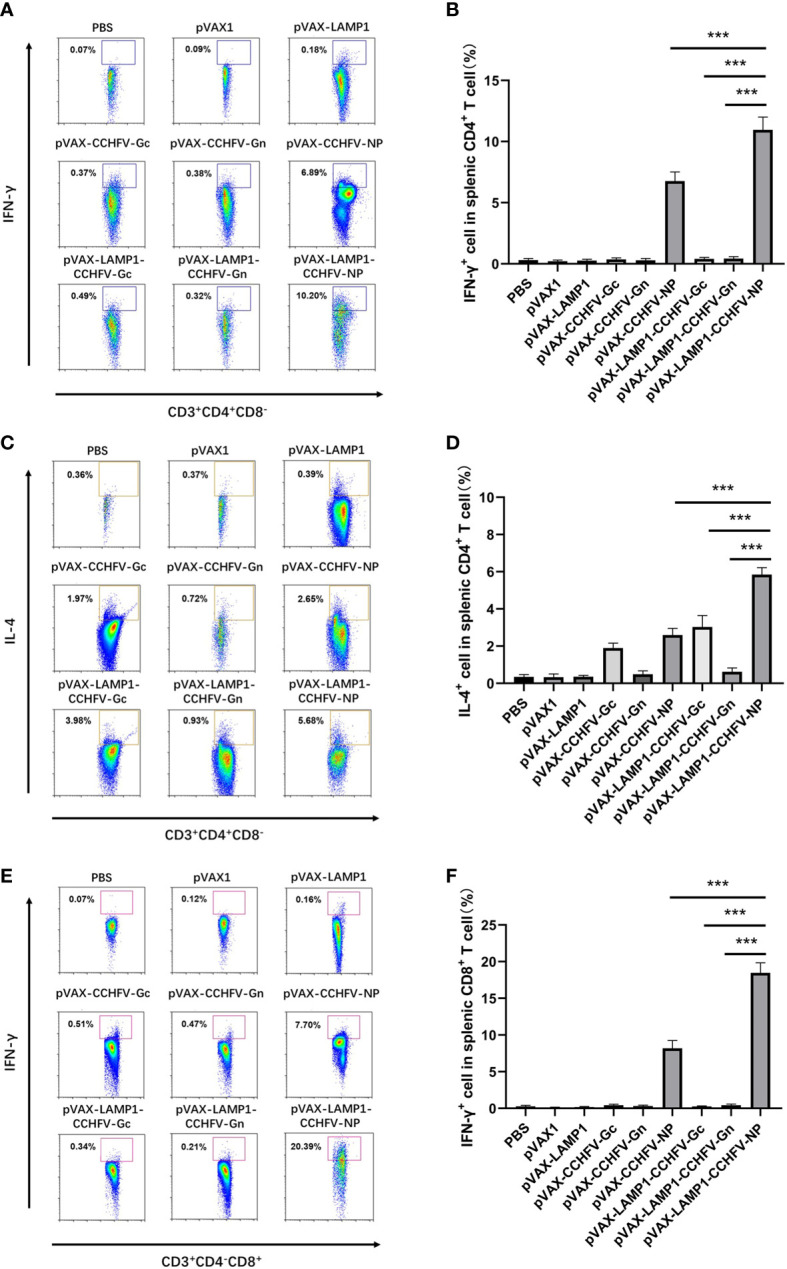
Identification of the types of T lymphocytes from the splenocytes of immunized mice. Mouse splenocytes were collected as described in the Materials and Methods section. The results are expressed as the mean value ± SD of five independent experiments. **(A)** Single-cell suspensions of splenocytes from immunized mice were gated with anti-CD3, anti-CD4, anti-CD8 and anti-IFN-γ antibodies. Representative flow cytometric plots are shown. **(B)** The percentage of IFN-γ+ cells in the CD4+ T cells from the spleens of immunized mice. **(C)** Single-cell suspensions of splenocytes from immunized mice were gated with anti-CD3, anti-CD4, anti-CD8 and anti-IL-4 antibodies. Representative flow cytometric plots are shown. **(D)** The percentage of IL-4+ cells in the CD4+ T cells from the spleens of immunized mice. **(E)** Single-cell suspensions of splenocytes from immunized mice were gated with anti-CD3, anti-CD4, anti-CD8 and anti-IFN-γ antibodies. Representative flow cytometric plots are shown. **(F)** The percentage of IFN-γ+ cells in the CD8+ T cells from the spleens of immunized mice. (*** P < 0.001).

To further evaluate the degree of immune activation in mice immunized with recombinant expression vectors, the cell type proportion of splenic lymphocytes was detected by flow cytometry. The mean levels of intracellular IFN-γ (10.96%) and IL-4 (5.85%) responses in CD4+ T cells from the mice immunized with pVAX-LAMP1-CCHFV-NP were higher compared to the pVAX-CCHFV-NP (p = 0.0003 for IFN-γ, p = 0.0005 for IL-4), pVAX-LAMP1-CCHFV-Gc (p = 0.0005 for IFN-γ, p = 0.0002 for IL-4) and pVAX-LAMP1-CCHFV-Gn groups (p = 0.0003 for IFN-γ, p = 0.0002 for IL-4) (**Figures 4A-D**). The mean levels of intracellular IFN-γ (18.47%) responses in CD8+ T cells from the mice immunized with pVAX-LAMP1-CCHFV-NP were also higher compared to the pVAX-CCHFV-NP (p = 0.0002), pVAX-LAMP1-CCHFV-Gc (p = 0.0003) and pVAX-LAMP1-CCHFV-Gn groups (p = 0.0005) (**Figures 4E, F**). These results demonstrated that pVAX-LAMP1-CCHFV-NP could induce strong CD8+ T-cell responses and balance Th1 and Th2 CD4+ T-cell responses, and pVAX-LAMP1-CCHFV-Gc mainly induced Th2 CD4+ T-cell responses. According to the experimental data of pVAX-LAMP1-CCHFV-NP and pVAX-LAMP1-CCHFV-Gc, the immune effect of pVAX-LAMP1-CCHFV-NP and pVAX-LAMP1-CCHFV-Gc was significantly improved compared with the vectors without the introduction of LAMP1. Moreover, pVAX-LAMP1-CCHFV-NP showed a more comprehensive ability to induce an immune response in the body, suggesting that the introduction of LAMP1 played an obvious role.

**Figure 5:**
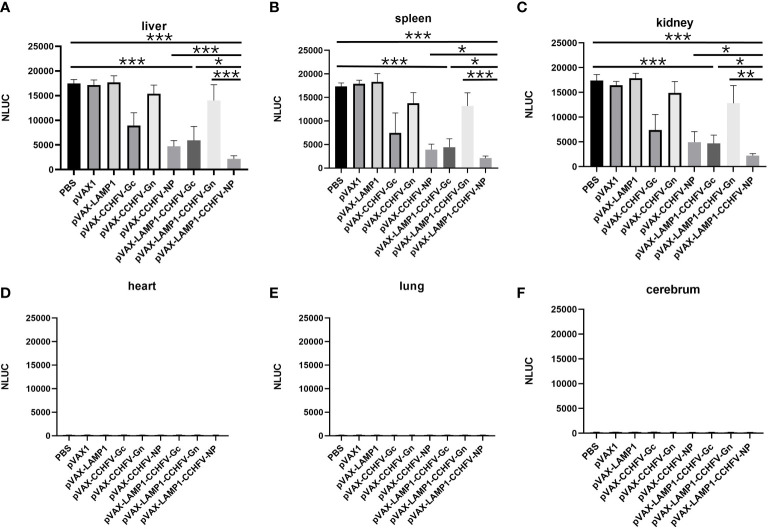
Detection of NanoLuc activity in the major tissues of immunized mice after challenge with CCHFV tecVLPs. The mouse tissues were collected as described in the Materials and Methods section. The results are expressed as the mean value ± SD of five independent experiments. **(A)** Detection of NanoLuc activities in the livers. **(B)** Detection of NanoLuc activities in the spleens. **(C)** Detection of NanoLuc activities in the kidneys. **(D)** Detection of NanoLuc activities in the hearts. **(E)** Detection of NanoLuc activities in the lungs. **(F)** Detection of NanoLuc activities in the cerebrum. (* P < 0.05, ** P < 0.01, *** P < 0.001).

### 3.5 CCHFV tecVLPs challenge of mice

To evaluate the *in vivo* protective effect of the recombinant expression vectors immunized mice, we attacked the immunized mice with CCHFV tecVLPs, and detected the content of CCHFV tecVLPs in major organs. The results showed that NanoLuc activities from the livers, spleens and kidneys of the mice immunized with pVAX-LAMP1-CCHFV-NP were much lower compared to mice injected with PBS (p = 0.0001 for livers, p = 0.0001 for spleens, p = 0.0005 for kidneys) and lower compared to pVAX-CCHFV-NP (p = 0.0001 for livers, p = 0.011 for spleens, p = 0.045 for kidneys), pVAX-LAMP1-CCHFV-Gc (p = 0.041 for livers, p = 0.042 for spleens, p = 0.032 for kidneys) and pVAX-LAMP1-CCHFV-Gn groups (p = 0.0009 for livers, p = 0.0007 for spleens, p = 0.003 for kidneys). These findings suggested that immunization with pVAX-LAMP1-CCHFV-NP provided much stronger protection from CCHFV tecVLPs challenge than any other group. The results also showed that NanoLuc activities from the livers, spleens and kidneys of the mice immunized with pVAX-LAMP1-CCHFV-Gc were lower compared to the PBS group (p = 0.0005 for livers, p = 0.0001 for spleens, p = 0.0003 for kidneys) (**Figures 5A–F**). This result indicated that immunization with pVAX-LAMP1-CCHFV-Gc also provided protection from CCHFV tecVLPs challenge, but the protective efficacies were less than those of pVAX-LAMP1-CCHFV-NP. The experimental data of pVAX-LAMP1-CCHFV-NP showed that the fusion expression of LAMP1 and NP could significantly improve the overall immune effect of the DNA vaccines, suggesting that LAMP1 could indeed play a role in promoting the immunogenicity of antigen proteins.

## 4 Discussion

The need for vaccines against CCHFV is urgent, particularly for populations at risk of exposure to CCHFV. In the current study, we used DNA plasmids expressing a LAMP1-linked version of CCHFV Gc, Gn and NP to immunize HLA-A11/DR1 mice. We demonstrate that mouse vaccination with pVAX-LAMP1-CCHFV-NP sufficiently elicited specific anti-NP antibodies and CTL responses in mice, and vaccination with pVAX-LAMP1-CCHFV-Gc mainly elicited specific anti-Gc and neutralizing antibodies. Moreover, pVAX-LAMP1-CCHFV-NP could most effectively protect mice from CCHFV tecVLPs infection, and pVAX-LAMP1-CCHFV-Gc also showed a certain protection from CCHFV tecVLPs infection in mice, but the protective efficacy was less than that of pVAX-LAMP1-CCHFV-NP. pVAX-LAMP1-CCHFV-Gn elicited only specific anti-Gn antibodies and could not provide sufficient protection from CCHFV tecVLPs infection.

The role of CCHFV NP in infection resistance and virus elimination remains unclear. However, many studies have shown that CCHFV NPs are produced in large quantities during infection and have high immunogenicity, containing B and T-cell epitopes (Goedhals et al., 2017; Moming et al., 2018). Recently, many research groups have used NP as an antigen to immunize knockout mice with complete protection against CCHFV infection (Aligholipour Farzani et al., 2019b; Aligholipour Farzani et al., 2019c). In these studies, NP-based vaccines induced powerful CTL responses in mice but low neutralizing antibody titers. These results are consistent with our study and indicate that neutralizing antibodies appear dispensable for vaccine-mediated protection, but specific CTL responses may be crucial for protection from CCHFV infection.

In addition to CTL responses, pVAX-LAMP1-CCHFV-NP sufficiently elicited higher anti-NP antibody titers than pVAX-CCHFV-NP. Although these antibodies do not have neutralizing activity, they can function in combination with NP to form immune complexes with antiviral activity. LAMP1, as a potential genetic adjuvant, plays an important role in the stronger CTL responses and higher anti-NP antibody titers induced by pVAX-LAMP1-CCHFV-NP. Our previous studies have shown that LAMP1 is desired for antigens directed to vesicular lysosomal/endosomal sites, where MHC II presentation occurs. After antigens are processed by the MHC II pathway, CD4+ T cells, which are critical for cellular and humoral immune responses, can be effectively primed. Moreover, by identification of the types of splenic T lymphocytes, CD4+ T cells from pVAX-LAMP1-CCHFV-NP exhibited a significantly higher number of IFN-γ and IL-4 spots than those from pVAX-CCHFV-NP (**Figure 4**). The results indicate that pVAX-LAMP1-CCHFV-NP could induce balanced Th1 and Th2 responses with the help of LAMP1. Many previous studies have confirmed that balanced Th1 and Th2 responses are also essential for protection in CCHFV-induced disease (Hinkula et al., 2017; Aligholipour Farzani et al., 2019c).

The glycoproteins Gn and Gc are largely considered preferred antigens for CCHFV vaccines, mainly because they are located on the surface of viral particles and thus are thought to be responsible for the induction of neutralizing antibodies. For this reason, monoclonal neutralizing antibodies against Gc have been described, but Gc-specific neutralizing antibodies do not provide sufficient protection, despite demonstrating *in vitro* viral neutralization (Bertolotti-Ciarlet et al., 2005; Golden et al., 2019). These results are also consistent with our study, but the detailed mechanism remains unknown. Moreover, by analyzing the cytokine responses in the spleens of immunized mice, CD4-positive splenocytes from pVAX-LAMP1-CCHFV-Gc immunized mice mainly exhibited a certain number of IL-4 secreting cells but not IFN-γ secreting cells. These results indicate that pVAX-LAMP1-CCHFV-Gc could not induce balanced Th1 and Th2 responses. To some extent, this explains the reasons for the poor protection effect of pVAX-LAMP1-CCHFV-Gc.

Progress in CCHFV vaccine research has been severely hampered by the lack of a suitable animal model. Previously, in addition to humans, the only other vertebrates known to be susceptible to CCHFV were newborn mice and rats (Smirnova, 1979; Shepherd et al., 1989). The immature immune system of these infant rodents is a major limitation of their use as an animal model. Recently, adult small animal models have been developed using mice deficient in the antiviral type I interferon (IFN) signaling pathway, either in the type I IFN receptor (IFNAR) (Bereczky et al., 2010; Hawman et al., 2021) or in STAT-1 (Bente et al., 2010). These immunocompromised mice after infection with CCHFV showed some disease signs and physiological changes experimentally, which is similar to the findings in humans, despite differences in the rate of disease onset and lethality. These immunocompromised mice have been successfully used for the evaluation of various CCHFV vaccines. However, the immune system of mice is quite different from that of humans, and the recognition of antigen epitopes is also very different. This is one of the reasons why many vaccines that have good effects on mouse models have poor effects when they enter clinical trials. Nonhuman primates (NHPs) may be potential animal models for CCHFV vaccines, but ethical and economic considerations clearly restrict research with NHPs (Haddock et al., 2018; Smith et al., 2019). The advent of humanized mice has opened up a new avenue for CCHFV vaccine research (Spengler et al., 2017). The human MHC molecule is the most polymorphic gene in the human genome. MHC restrictions vary significantly by geographical region and race. Some studies have used MHC-I or MHC-II transgenic mice to evaluate candidate vaccines and screen HLA-restricted epitopes. We successfully obtained a novel human MHC (HLA-A11/DR1) transgenic mouse model expressing both HLA-A11 and HLA-DR1 molecules in the absence of H-2 class I/II molecules in cooperation with the State Key Laboratory of Pathogen and Biosecurity (Beijing, China) in the early stage (Zeng et al., 2016). HLA-A11/DR1 mice showed the combined expression of HLA-I allele a11 and HLA-II allele DR1, which are common among the world population (Lee et al., 1988). This new animal model has been successfully used for the evaluation of Ebola virus (EBOV) (Li et al., 2019) and hepatitis B virus (HBV) vaccines (Zeng et al., 2016) and will also contribute to the establishment of an animal evaluation model for CCHFV vaccines.

Progress with CCHFV vaccine research has also been severely hindered by the requirement of high-containment laboratories to handle the virus. CCHFV infection in cells and animals requires biosafety level 4 (BSL-4) facilities (Hinkula et al., 2017). This limits the extensive development of CCHFV vaccine research. We successfully constructed the CCHFV tecVLPs system and established a Huh7 cell infection model and HLA-A11/DR1 mouse infection model (**Supplementary Figures 2–5**). TecVLPs are composed of viral proteins and minigenomes that together form a virus-like particle with the same morphology as CCHFV. They can mimic the viral replication cycle, including viral cell entry and subsequent transcription, without producing infectious virus (Zivcec et al., 2017). Therefore, we can infect cells or mice to evaluate the neutralizing antibody titer or animal protective efficacy of CCHFV tecVLPs under BSL-2 conditions. In summary, our data demonstrate that pVAX-LAMP1-CCHFV-NP could induce higher anti-NP antibody titers, stronger CTL responses and more sufficient protective efficacy in mice than any other vaccine. The results suggest that pVAX-LAMP1-CCHFV-NP is a potential and powerful candidate vaccine for CCHFV. However, there are several important limitations of our study that will require further investigation. First, we used CCHFV to test the neutralizing antibody titer *in vitro* and the protective efficacy *in vivo* of the vaccines to verify the accuracy of using tecVLPs to replace the virus for vaccine evaluation. It has been reported that CCHFV tecVLPs can be used to screen neutralizing antibodies *in vitro* (Zivcec et al., 2017), but this is the first study to evaluate the protective efficacy of a vaccine *in vivo*. Second, it is necessary for us to carry out long-term immune experiments and safety evaluations of the vaccine in the future, which will help to carry out clinical research and obtain an application license for the vaccine.

In this study, we constructed three recombinant expression vectors containing the LAMP1 sequence to express the LAMP1 and CCHFV antigen fusion proteins. LAMP1 acted as an immune adjuvant. In previous studies, Aligholipour Farzani et al. immunized mice with a DNA recombinant expression vector expressing CCHFV NP protein while co-immunizing a DNA recombinant expression vector expressing CD24. CD24 acted as a potential immune adjuvant (Aligholipour Farzani et al., 2019c). CD24 is a highly glycosylated mucin-like cell surface protein present in B and T lymphocytes, neutrophils, and macrophages that stimulates the expansion of B lymphocytes and CD4+ T lymphocytes. The results of the above research showed that NP co-expression with CD24 could significantly improve the specific immune response of immunized mice, and the levels of humoral immunity and cellular immunity were significantly increased. These results are consistent with the trend of our research results. At the same time, the experimental results after CCHFV challenge showed that the survival rate of mice in the NP and CD24 co-expression group was 100%, indicating that the introduction of appropriate adjuvants at the same time of DNA vaccine immunization can significantly improve the immune protection effect.

While for the application of tecVLP, tecVLP was basically used as a reverse genetics tool to detect neutralizing antibody titers in serum of immunized animals. In this study, we used CCHFV tecVLP as an alternative virus to attack animals, which is innovative for tecVLP application. On the other hand, although tecVLP has a complete viral envelope, it is still not a live virus. It lacks the genome of wild-type virus and is unable to complete intracellular self-replication, so it has some defects in simulating intracellular replication of virus. At the same time, because tecVLP does not have a complete viral genome, its safety is guaranteed, and it can be used as a virus substitute in low-level laboratories. In the future, we will further use CCHFV virus for animal protection experiments to further verify the animal protection effect of mice in each immune group and compare the protective effect with that of CCHFV tecVLP after infection. At the same time, we still have much work to do on the optimization and perfection of the CCHFV tecVLPs infection model, and there are many details to be solved. In order to better promote and apply tecVLP in the future, it is necessary to further confirm the rationality of the application of CCHFV tecVLP as a substitute for the virus in vaccine evaluation.

## Data availability statement

The original contributions presented in the study are included in the article/**Supplementary Material**. Further inquiries can be directed to the corresponding authors.

## Ethics statement

The animal study was reviewed and approved by Air Force Medical University Animal Experimental Welfare & Ethical Committee (Xi’an, Shaanxi, China; approval no. 20210396).

## Author contributions

L-FC and F-LZ developed the original idea. L-FC, F-LZ, and D-BJ designed the whole study. D-BJ, Y-LH, Z-XQ, and L-XZ constructed the plasmids. Y-LH and X-QL performed the IF and WB experiments. Y-LH, X-QL, Y-XZ, Z-XQ, and L-XZ evaluated the immune responses. L-QZ, WY, LZ, Y-FL, and X-QL carried out the CCHFV tecVLPs challenges. Y-LH, X-QL, Y-XZ, Z-XQ, LZ, and L-XZ took care of the animals. L-FC and Y-LH processed the data and calculated the statistics. Y-LH, L-FC, F-LZ, and D-BJ drafted the manuscript. All authors contributed to the article and approved the submitted version.
