# Supplementary material for: Construction and evaluation of DNA vaccine encoding Crimean Congo hemorrhagic fever virus nucleocapsid protein, glycoprotein N-terminal and C-terminal fused with LAMP1
Source: Front Cell Infect Microbiol. 2023 Mar 21;13:1121163. doi: 10.3389/fcimb.2023.1121163 (PMC10072157; doi:10.3389/fcimb.2023.1121163)
Supplement: Supplementary file 1 [file DataSheet_1.docx]

Supplementary Material

**Construction and evaluation of DNA vaccine encoding Crimean Congo Hemorrhagic Fever Virus Nucleocapsid Protein, glycoprotein N-terminal and C-terminal fused with LAMP1**

Yong-Liang Hu^1, 2#^, Lian-Qing Zhang^1, 3#^, Xiao-Qian Liu^1, 4#^, Wei Ye^1^, Yue-Xi Zhao^1, 4^, Liang Zhang^1^, Zun-Xian Qiang^1^, Lin-Xuan Zhang^1^, Ying-Feng Lei^1^, Dong-Bo Jiang^5*^, Lin-Feng Cheng^1*^, Fang-Lin Zhang^1*^

^1^Department of Microbiology, Air Force Medical University (The Fourth Military Medical University), Xi’an, 710032, China

^2^Department of Dermatology, The eighth medical center of PLA general hospital, Beijing, 100091, China

^3^College of Life Sciences, Northwest University, Xi’an, 710069, China

^4^School of Medical Technology, Shaanxi University of Chinese Medicine, Xianyang, 712046, China

^5^Department of Immunology, Air Force Medical University (The Fourth Military Medical University), Xi’an, 710032, China

^#^ Yong-Liang Hu, Lian-Qing Zhang, Xiao-Qian Liu contributed equally to this work.

* Correspondence:

Dong-Bo Jiang

[superjames1991@foxmail.com](mailto:superjames1991@foxmail.com)

Lin-Feng Cheng

[chenglfz@fmmu.edu.cn](mailto:chenglfz@fmmu.edu.cn)

Fang-Lin Zhang

[flzhang@fmmu.edu.cn](mailto:flzhang@fmmu.edu.cn)

# Supplementary Figures and Tables

## Supplementary Figures

Supplementary Figure 1. Plasmid map of pVAX-LAMP1.


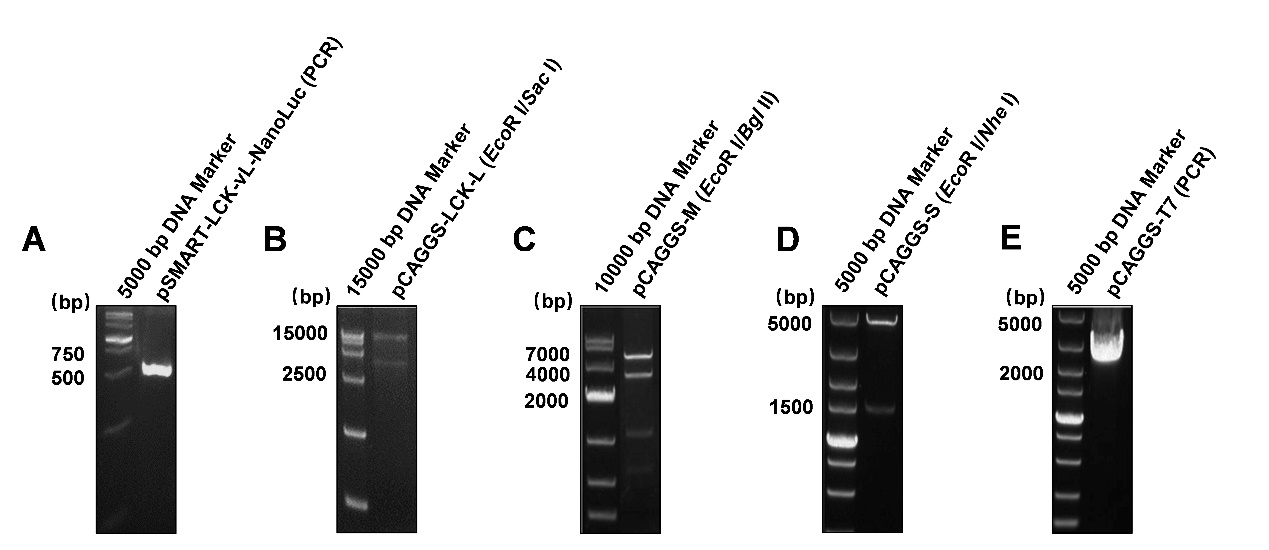


Supplementary Figure 2. Identification of recombinant plasmids for the production of CCHFV tecVLPs by PCR and restriction enzyme digestion.

Recombinant plasmids encoding CCHFV-specific NanoLuc minigenome (pSMART-LCK-vL-NanoLuc), CCHFV RdRP (pCAGGS-LCK-L), GP (pCAGGS-M), NP (pCAGGS-S) and T7 polymerase (pCAGGS_T7) were constructed and identified by PCR and restriction enzyme digestion. (A) Identification of pSMART-LCK-vL-NanoLuc by PCR. (B) Identification of pCAGGS-LCK-L by restriction enzyme digestion. (C) Identification of pCAGGS-M by restriction enzyme digestion. (D) Identification of pCAGGS-S by restriction enzyme digestion. (E) Identification of pCAGGS-T7 by PCR.


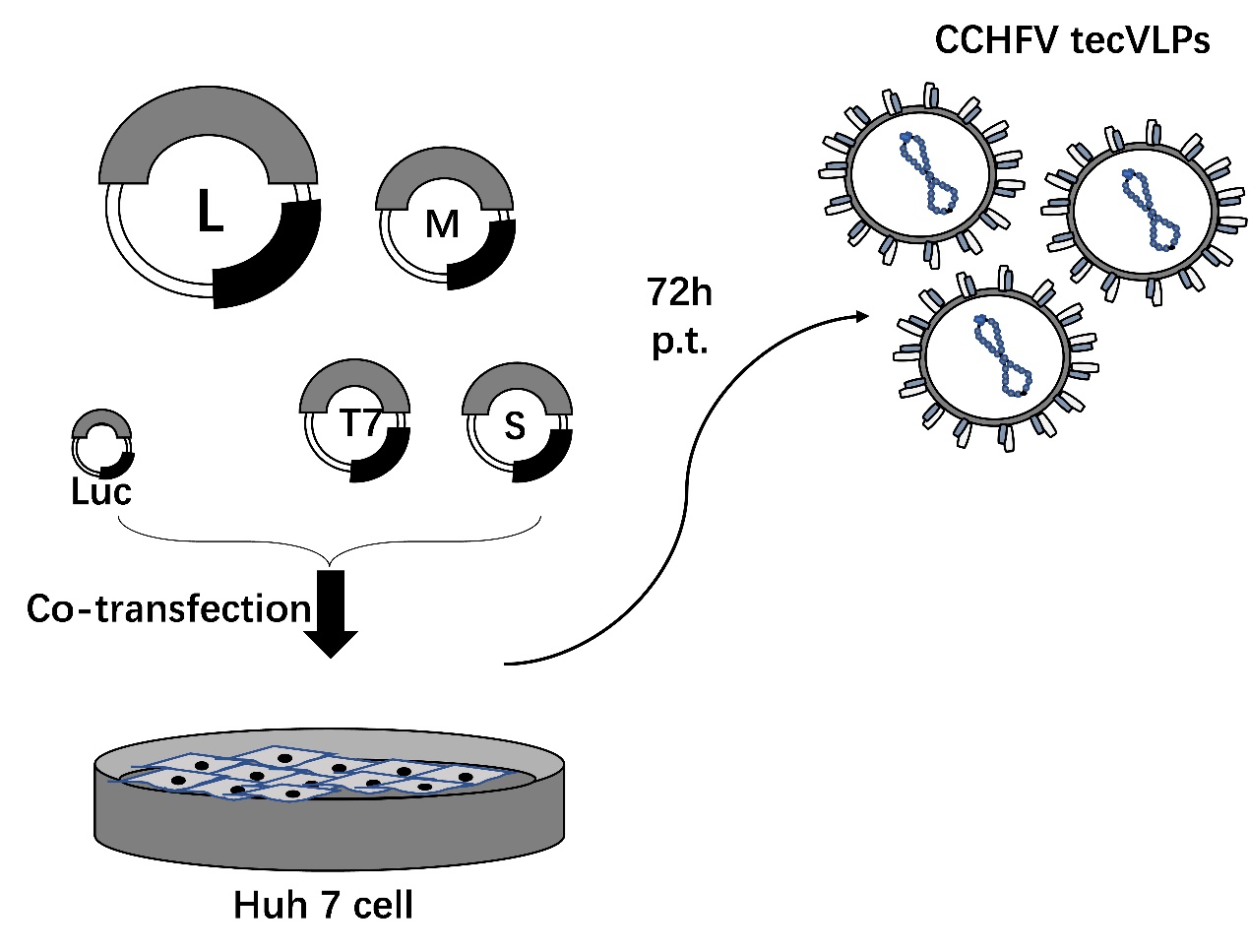


Supplementary Figure 3. Production of CCHFV tecVLPs

General outline of the procedure to generate CCHFV tecVLPs. Subconfluent monolayers of Huh 7 cells seeded in 6-well plates were cotransfected with 100 ng of pSMART-LCK-vL-Nanoluc, 800 ng of pCAGGS-LCK-L, 400 ng of pCAGGS-M, 400 ng of pCAGGS-S and 200 ng of pCAGGS-T7. Cell supernatants were collected 72 h posttransfection, centrifuged at 12,000 × g for 5 min to remove cellular debris and filtered through a 0.45 µm pore-size filter. Next, the tecVLPs were concentrated via ultrafiltration using a 15-ml centrifugal filter with a 3 kDa cut-off (Millipore, Billerica, MA, USA) at 3000 rpm for 2 h at 4 ℃ in a Thermo Scientific 75003608 Rotor (Waltham, MA, USA).

Supplementary Figure 4. Establishment of a cell infection model of CCHFV tecVLPs

(A) General outline of the procedure to establish the cell infection model of CCHFV tecVLPs. Huh 7 cells seeded in 96-well plates were cotransfected with 50 ng of pCAGGS-LCK-L and 25 ng of pCAGGS-S 24 h before 100 TCID_50_ CCHFV tecVLPs infection. Three days after CCHFV tecVLPs infection, the cells were collected, lysed and then centrifuged at 12,000 rpm for 30 min at 4 ℃. The supernatants were collected and detected using the Nano-Glo™ Luciferase Assay System to establish a cell infection model. (B) Detection of NanoLuc activities from the cells using the Nano-Glo™ Luciferase Assay System. Control: normal Huh 7 cells; L+S: Huh 7 cells cotransfected with pCAGGS-LCK-L and pCAGGS-S; TecVLPs: Huh 7 cells infected with CCHFV tecVLPs; L+S+TecVLPs: Huh 7 cells cotransfected with pCAGGS-LCK-L and pCAGGS-S and then infected with CCHFV tecVLPs. The results are expressed as the mean value ± SD of five independent experiments. (**P < 0.01)

Supplementary Figure 5. Establishment of an animal CCHFV tecVLPs infection model

(A) General outline of the procedure to establish an animal infection model of CCHFV tecVLPs. HLA-A11/DR1 mice were coinjected with 20 µg of pCAGGS-LCK-L and 10 µg of pCAGGS-S 24 h before 100 TCID_50_ CCHFV tecVLPs infection. Three days after CCHFV tecVLPs infection, the mice were killed by cervical dislocation, and tissues (including liver, kidney, spleen, lung, cerebrum and heart) were collected and weighed from these mice, diluted in PBS, and then freeze‒thawed (-80/37°C) three times after being ground to prepare 10% (g/mL) tissue suspensions. The samples were centrifuged at 12,000 rpm for 30 min at 4 ℃, and the supernatants were collected. Then, Nano-Luc activities were detected using the Nano-Glo™ Luciferase Assay System to establish an animal infection model. (B) Detection of NanoLuc activities from the tissues using the Nano-Glo™ Luciferase Assay System. Control: normal HLA-A11/DR1 mice; L+S: HLA-A11/DR1 mice coinjected with pCAGGS-LCK-L and pCAGGS-S; TecVLPs: HLA-A11/DR1 mice infected with CCHFV tecVLPs; L+S+TecVLPs: HLA-A11/DR1 mice preinjected with pCAGGS-LCK-L and pCAGGS-S and then infected with CCHFV tecVLPs. The results are expressed as the mean value ± SD of five independent experiments. (**P < 0.01, *** P <0.001)

## Supplementary Tables

Supplementary Table 1. List of primers

| Primers | Sequence | |  |
| --- | --- | --- | --- |
| pVAX-LAMP1-CCHFV-NP (forwards)  pVAX-LAMP1-CCHFV-NP (reverse)  pVAX-CCHFV-NP (forwards)  pVAX-CCHFV-NP (reverse)  pVAX-LAMP1-CCHFV-Gc (forwards)  pVAX-LAMP1-CCHFV-Gc (reverse)  pVAX-CCHFV-Gc (forwards)  pVAX-CCHFV-Gc (reverse)  pVAX-LAMP1-CCHFV-Gn (forwards)  pVAX-LAMP1-CCHFV-Gn (reverse)  pVAX-CCHFV-Gn (forwards)  pVAX-CCHFV-Gn (reverse) | | 5′-CTGCTGGACGAGAACAGCATGGAAAACAAGATCGAGG-3′  5′-CAGCGATGGGGATCAGCATAATGATGTTAGCACTGGTGG-3′  5′-TCCAGTGTGGTGGAATTCATGGAAAACAAGATCGAGGTG-3′  5′-TTAAACGGGCCCTCTAGATTAAATGATGTTAGCACTG-3′  5′-CTGCTGGACGAGAACAGCTTCCTGGACAGCACCGCCA-3′  5′-CAGCGATGGGGATCAGCATCCCGATGTGGGTCTTTGTGG-3′  5′-TCCAGTGTGGTGGAATTCATGTTCCTGGACAGCAC-3′  5′-TTAAACGGGCCCTCTAGATCACTACCCGATGTGGGT-3′  5′-CTGCTGGACGAGAACAGCAGTGAGGAACCATCAGACG-3′  5′-CAGCGATGGGGATCAGCATGATTGGAGCGGACTGCAC-3′  5′-TCCAGTGTGGTGGAATTCATGAGTGAGGAACCAT-3′  5′-TTAAACGGGCCCTCTAGACTAGATTGGAGCGGACTG-3 ′ | |

Supplementary Table 2. Schedule for animal experiments

| Groups | Amount of animals | Immunizing dose/time | Post-immunization testing |
| --- | --- | --- | --- |
| pVAX-LAMP1-CCHFV-NP  pVAX-LAMP1-CCHFV-Gc  pVAX-LAMP1-CCHFV-Gn  pVAX-CCHFV-NP  pVAX-CCHFV-Gc  pVAX-CCHFV-Gn  pVAX-LAMP1  pVAX  PBS | 10 mice per group | 70μg/200μl/mouse  70μg/200μl/mouse  70μg/200μl/mouse  70μg/200μl/mouse  70μg/200μl/mouse  70μg/200μl/mouse  70μg/200μl/mouse  70μg/200μl/mouse  200μl/mouse | ①3 weeks after the last immunization, 5 mice from each group were sacrificed and sera and splenic cells were collected. Specific antibodies detection, neutralizing antibodies detection, secretion of cytokines detection (ELISPOT), splenocytes cytotoxic activity detection (CTL) and splenic lymphocyte typing detection (flow cytometry) were performed.  ②The remaining 5 mice in each group were challenged with CCHFV tecVLP. After 3 days, the mice were sacrificed to detect the CCHFV tecVLP load in each major organ |
